# Patient Safety Culture: Current Status and Associated Factors Among Healthcare Workers at Kien An Hospital—Hai Phong, Vietnam—A Cross-Sectional Study

**DOI:** 10.3390/ijerph23081064

**Published:** 2026-08-17

**Authors:** Nguyen Ba Phuoc, Vu Tuan Anh, Nguyen Thi Ly, Nguyen Duc Son, Nguyen Thi Chin, Lam Thi Hanh, Trinh Thi My, Nguyen Thi Nhan, Le Van Mang, Luu Xuan Quy, Ha Thi Minh Nguyet, Nguyen Duc Thanh

**Affiliations:** 1 Kien An Hospital, Hai Phong 180000, Vietnam; nguyenbatuankiet@gmail.com (N.B.P.); vutuananh1312@gmail.com (V.T.A.); nguyenlycka@gmail.com (N.T.L.); nguyenducsonktmhp@gmail.com (N.D.S.); huyenchin75@gmail.com (N.T.C.); lamhanh90hp@gmail.com (L.T.H.); trinhmy.bvka@gmail.com (T.T.M.); mhm2130016@studenthuph.edu.vn (N.T.N.); 2 Medical Affairs Division, Department of Health, Hai Phong 180000, Vietnam; lvmangsyt@gmail.com; 3 Health Management Training Institute, Hanoi University of Public Health, Hanoi 11910, Vietnam; lxq@huph.edu.vn (L.X.Q.); ndt@huph.edu.vn (N.D.T.)

**Keywords:** patient safety culture, healthcare staff, hospital, physicians, nurses

## Abstract

**Highlights:**

**Public health relevance—How does this work relate to a public health issue?**

- Preventable medical incidents pose a significant threat to both patient well-being and the systemic integrity of the healthcare infrastructure.
- Measuring and understanding patient safety culture (PSC) is globally recognized as an essential, foundational step toward enhancing the quality and safety of healthcare delivery.

**Public health significance—Why is this work of significance to public health?**

- This research provides critical, evidence-based insights into the localized strengths and vulnerabilities of safety culture within a Vietnamese public hospital setting.
- The study reveals severe dual vulnerabilities in psychological safety: “Communication Openness” recorded the absolute lowest positive response rate at 25.9%, closely followed by “Non-punitive response to error” at 29.0%, pointing to a pervasive “blame culture” that suppresses open dialogue.

**Public health implications—What are the key implications or messages for practitioners, policy makers and/or researchers in public health?**

- Hospital administrators and policymakers must urgently prioritize cultivating psychological safety by decoupling error reporting from administrative or financial penalties to encourage voluntary reporting.
- Multivariable analysis demonstrates that safety culture perceptions are heavily driven by age rather than professional role or department; therefore, targeted safety initiatives and onboarding protocols must be urgently tailored to support early-career healthcare workers (under 30 years old) who view the safety climate least favorably.

**Abstract:**

**Objective**: Patient safety culture (PSC) is a critical determinant of healthcare quality. This study aimed to assess the current status of PSC among healthcare workers at Kien An Hospital, Hai Phong, Vietnam, and identify associated factors. **Methods**: A cross-sectional survey was conducted using the 12-dimension hospital survey on patient safety culture (HSOPSC). A total of 324 healthcare professionals (physicians, nurses, and allied staff) participated. Data were analyzed using descriptive statistics and multivariable logistic regression. **Results**: The positive response rate (PRR) across the 12 dimensions of patient safety culture (PSC) varied widely. The highest positive perceptions were observed in “Teamwork within units” (98.5%) and “Teamwork across units” (98.1%). Conversely, severe vulnerabilities in psychological safety were identified, with “Communication Openness” recording the absolute lowest PRR at 25.9%, closely followed by “Non-punitive response to error” at 29.0%. Comparative analysis between professional strata revealed broad alignment across 11 dimensions, with the sole significant divergence occurring in “Feedback and communication about error”, where physicians reported a significantly higher PRR than nurses & allied staff (85.8% vs. 75.2%; *p* = 0.04). In the multivariable logistic regression model, age emerged as the single independent predictor of overall positive PSC; compared to early-career staff (<30 years), healthcare workers aged 30–40 years (AOR = 4.17, *p* = 0.02) and 40–50 years (AOR = 5.03, *p* = 0.02) had significantly higher odds of favorable safety perceptions. **Conclusions**: Healthcare workers at the study hospital exhibit exceptionally strong collaborative teamwork, yet low communication openness and the fear of a punitive environment remain critical systemic barriers to safety. Because safety culture challenges are macro-institutional and disproportionately impact junior personnel, interventions should shift away from role-specific policies. Instead, hospital leadership must prioritize establishing non-punitive reporting mechanisms, enhancing open dialogue, and designing targeted safety culture onboarding initiatives tailored specifically to the needs of early-career healthcare workers to foster continuous organizational learning.

## 1. Introduction

Patient safety culture (PSC) fundamentally reflects the shared values, attitudes, perceptions, and behavioral patterns that determine a healthcare facility’s commitment to safety management to reduce adverse events which remain a critical global health challenge. According to the World Health Organization (WHO), patient harm is the 14th leading cause of the global disease burden, with approximately 1 in 10 patients harmed while receiving hospital care in high-income countries. In low- and middle-income countries, the prevalence is even more concerning, contributing to approximately 134 million adverse events annually due to unsafe care [1]. Adverse events during hospital delivery represent a major threat to global public health, exhibiting distinct prevalence variations based on regional economic structures and safety maturity. According to epidemiological guidelines compiled by WHO, patient harm stands as one of the leading causes of the global disease burden. In high-income countries, the prevalence rate of adverse events is established at approximately 10%, meaning 1 in every 10 hospitalized patients encounters some form of preventable medical harm. Data published in the National Scorecard on Hospital-Acquired Conditions by the Agency for Healthcare Research and Quality (AHRQ) highlights that while targeted tracking initiatives have driven down specific hospital-acquired conditions by up to 13% in mature settings, the baseline risk vectors remain persistently present within active clinical workflows [2].

Measuring PSC is globally recognized as an essential, foundational step toward identifying systematic vulnerabilities and improving healthcare quality. In Vietnam, the implementation of patient safety processes is legally mandated through several regulatory frameworks issued by the Ministry of Health. Specifically, Circular No. 19/2013/TT-BYT requires hospitals to establish quality management systems, and Circular No. 43/2018/TT-BYT focuses on medical incident prevention and reporting. Although these regulations align with many principles recommended by the AHRQ, such as the establishment of specialized safety committees, the practical integration of a non-punitive safety culture remains inconsistent across different hospital levels, often hindered by traditional hierarchical structures [3]. Nurses and medical technicians submitted 60.2% of all medical incident reports. Practice-related errors by healthcare professionals accounted for 52.04% of reported incidents. Root cause analysis (RCA) was performed for 76.53% of incidents, and 51.02% were classified as recurrent events [4].

Patient safety within healthcare organizations garnered considerable attention following the seminal 1999 Institute of Medicine’s report, “To Err is Human: Building a Safer Health System” [5]. Nieva et al. considered PSC as the product of individual and group values, attitudes, perceptions, competencies, and patterns of behavior that determine the commitment to, and the proficiency of, an organization’s health and safety management. A truly mature safety culture is characterized by organizational openness and fairness toward staff following adverse events, a demonstrated willingness to learn from mistakes, and a clear focus on identifying and correcting system failures rather than assigning individual blame [6].

PSC fundamentally refers to how safety is managed and perceived in the workplace, reflecting the shared attitudes, beliefs, perceptions, and values of employees regarding safety [7]. The WHO defines patient safety as the reduction of unnecessary risk and harm associated with healthcare to an acceptable minimum. PSC is a complex framework encompassing multiple dimensions that collectively influence a wide range of discretionary behaviors related to patient safety [6]. According to AHRQ [8], PSC involves understanding the values, beliefs, and norms about what is important within an organization, as well as recognizing which attitudes and behaviors related to patient safety are supported, rewarded, and expected. Measuring patient safety culture is widely regarded as the crucial first step toward enhancing the quality of healthcare delivery. Therefore, healthcare providers are increasingly urged to make PSC a top priority in their organizational practices [9].

The study utilized the Hospital Survey on Patient Safety Culture (HSOPSC, a validated instrument initially developed by AHRQ in the United States and widely applied across numerous countries [8]. This comprehensive instrument is designed to measure 12 dimensions of PSC categorized across different organizational levels, including 7 dimensions at the unit level that focus on teamwork, communication, and management support within the clinic unit; 3 dimensions at the hospital level addressing hospital-wide teamwork, staffing, and organizational learning; and 2 outcome dimensions capturing the overall perception of safety and the frequency of reported events [8]. The HSOPSC tool has been previously translated, subjected to reliability validation, and successfully utilized in assessing PSC across various hospital settings throughout Vietnam [10].

While safety culture research has grown globally, empirical evidence from provincial general hospitals in Vietnam remains limited. This study aimed to (1) evaluate the status of PSC among healthcare workers at Kien An Hospital, Hai Phong, Vietnam using the HSOPS-VN; and (2) identify personal and professional factors independently associated with overall positive PSC perceptions.

## 2. Methods

### 2.1. Study Design and Setting

This was a cross-sectional study conducted from 1 May to 20 June 2025 at Kien An Hospital, Hai Phong, Vietnam. Kien An Hospital is a public, Grade-I provincial general hospital serving as a tertiary regional referral center. It has an official bed capacity of 600 beds and employs approximately 550 total personnel across clinical, para-clinical, and administrative departments.

### 2.2. Study Population and Sampling

The study population comprised full-time healthcare professionals (physicians, nurses, midwives, technicians, and pharmacists).

Inclusion criteria: (1) Employed full-time; (2) at least 6 months of experience; and (3) voluntary participation.

Exclusion criteria: Healthcare workers were attending off-site training courses, on business trips, or on maternity leave during the data collection period.

Sampling procedure and participants: A census sampling approach was deployed. Out of 360 eligible healthcare workers invited across hospital departments during the study window, 332 completed the questionnaire, and 8 were excluded due to incomplete responses (>20% missing items), yielding a final analyzed cohort of *n* = 324 (Response rate = 90.0%). Under this framework, the entire accessible population of 324 healthcare workers who strictly fulfilled the baseline inclusion criteria was used for analysis. Using a census approach for all eligible personnel minimized selection bias and provided a comprehensive representation of the hospital’s safety climate.

To ensure privacy and minimize self-report bias (STROBE 8), the survey was conducted under strict confidentiality protocols and was completely anonymous. In addition, to maintain confidentiality, participants were instructed to place their completed questionnaires in sealed envelopes. The study coordinators were responsible for collecting these sealed envelopes and transferring them directly to the research team for data entry and analysis.

### 2.3. Measurement Instrument and Internal Consistency:

PSC was evaluated using the official Vietnamese version of the HSOPS-VN (details questionnaire in Appendix A), which corresponds to the 42-item, 12-dimension framework of the original AHRQ HSOPSC Version 1 [8,9]. To maximize psychometric reliability and maintain methodological consistency with established national baselines, the research team deliberately chose not to execute an independent, ad-hoc translation. Instead, we applied this pre-validated tool, which had historically undergone strict cross-cultural adaptation protocols. This adaptation previously resolved the profound complexities of the Vietnamese language—specifically its reliance on six distinct phonetic tones and intricate diacritical marks where subtle variations in syllabic accents can alter the interpretation of written clinical texts. Prior large-scale psychometric studies within Vietnamese public provincial and specialized hospitals have firmly established the tool’s strong structural validity and high internal consistency (Cronbach’s alpha ranging from 0.68 to 0.88 across dimensions), with the highest reliability observed in Frequency of Events Reported (0.85) and Teamwork Within Units (0.83), reflecting strong item cohesion. Conversely, the Staffing domain recorded the lowest internal consistency score at 0.62, making it the only domain below the standard 0.70 reliability threshold [8,10].

Scoring Mechanism: Every item on the survey was scored using a standardized 5-point Likert scale measuring either agreement (1 = Strongly Disagree to 5 = Strongly Agree) or operational frequency (1 = Never to 5 = Always). The baseline metric calculated for each dimension was the Positive Response Rate (PRR, representing the percentage of favorable answers. For the 34 positively worded items, responses of ‘Agree/Strongly Agree’ or ‘Often/Always’ were classified as positive (Scores 4 and 5). For the 8 negatively worded items, a reverse-scoring protocol was executed; thus, responses of ‘Disagree/Strongly Disagree’ or ‘Never/Rarely’ were mathematically inverted and classified as positive (Scores 1 and 2 converted to positive indicators). Responses indicating neutrality (‘Sometimes’ or ‘Neutral’) were uniformly categorized as non-positive. The 12 dimensions were grouped into unit-level factors, hospital-level factors, and clinical outcome domains as detailed below:

(a) Unit-level (7 dimensions): Supervisor/manager expectations promoting safety (4 items); Organizational learning—continuous trainings (3 items); Teamwork within units (4 items); Communication openness (3 items); Feedback and communication about error (3 items); Non-punitive response to error (3 items); Staffing (4 items).
(b) Hospital-level (3 dimensions): Hospital management support for safety (3 items); Teamwork across units (4 items); Handoffs and transitions (4 items).
(c) Outcome-level (2 dimensions): Overall perceptions of patient safety (4 items); Frequency of event reporting (3 items).

Internal Consistency: The reliability of the AHRQ data, as measured by Cronbach’s α, ranges from 0.63 to 0.84 [8]. In the present study, the Cronbach’s α values for the 12 dimensions ranged from 0.62 to 0.82 [10]. While this demonstrates acceptable internal consistency, the slightly lower values compared to the AHRQ reference data suggest a marginally lower internal consistency of responses within this specific cohort.

Data cleaning and processing strictly adhered to the standardized evaluation methodologies established by the Agency for Healthcare Research and Quality (AHRQ). Prior to executing descriptive and multivariable analyses, a rigorous reverse-coding protocol was implemented for all negatively worded items within the HSOPS-VN instrument. Specifically, for these negative indicators, responses of ‘Disagree’ or ‘Strongly Disagree’ (and ‘Rarely’ or ‘Never’) were mathematically inverted to represent a favorable safety perception.

Following this transformation, the PRR was calculated for each individual dimension and combined into a macro-level overall safety culture metric. This methodological adherence ensures that our baseline safety percentages are highly reliable and directly comparable to both regional and international public health databases.

Although patient safety culture predominantly impacts frontline clinical personnel, non-direct care staff (such as support, administrative, and para-clinical personnel) also operate within and influence the hospital’s overarching safety ecosystem. Therefore, ‘direct patient contact’ (Yes/No) was retained as a controlling variable in multivariable logistic regression analyses. This allowed the model to isolate whether direct daily exposure to patient care independently affects safety perceptions, while statistically controlling for potential confounding introduced by varying operational proximity to direct clinical risks.

### 2.4. Data Analysis

Data analysis was executed using SPSS version 20.0 (IBM Corp., Armonk, NY, USA). Initially, descriptive statistics summarized participant demographics and calculated the PRR for each individual HSOPSC dimension. To evaluate differences in the proportions of positive safety culture perceptions between professional groups (Physicians versus Nurses & Allied Staff), Chi-square tests for independence were deployed. For cell counts falling below expected statistical frequencies, Fisher’s exact tests were substituted. Subsequently, to identify independent predictors of an optimal safety climate, multivariable logistic regression modeling was utilized. The dependent variable, ‘Overall Perception of Patient Safety Culture,’ was constructed as a dichotomous outcome derived from the cumulative scoring of all 42 survey items. For each participant, an overall individual percent positive score was calculated by dividing the sum of their positive item responses (Likert scores of 4 or 5 on positively worded items, and reverse-scored 1 or 2 on negatively worded items) by the total number of items. The study health staff grouping was based on similar workflow structures (shift-based clinical care execution) vs. autonomous medical decision-making (physicians) for analysis.

Based on the official benchmarking criteria defined by AHRQ, a rigid cut-off threshold of ≥75% was established to categorize a participant’s perspective as a ‘Positive PSC Perception’. Applying this threshold to individual respondents’ total percent positive score is an established adaptation in epidemiological survey research [8,11]. Respondents whose cumulative positive score fell below this 75% threshold were categorized as ‘Not Positive’. Under AHRQ parameters, a score of 75% or higher formally designates an institutional domain or climate as an organizational ‘safety culture strength’. This allows dichotomization into “Positive” vs. “Not positive” overall perceptions for multivariable logistic regression.

The decision to dichotomize this multidimensional construct—rather than analyzing scores on a purely continuous scale—was mathematically and operationally preferred for three reasons: first, it directly aligns the study’s predictive metrics with standardized AHRQ international reporting baselines; second, it translates abstract continuous data into high-impact, actionable administrative categories for hospital quality management teams; and third, it permits the calculation of multivariable Adjusted Odds Ratios (aOR). The variables were selected based on a combination of theoretical/clinical relevance (as established confounding variables in safety culture literature) and univariable screening. Prior to executing the multivariable logistic regression model, multicollinearity among predictor variables (particularly highly correlated demographic variables such as age and years of service) was evaluated using Variance Inflation Factors (VIF) and tolerance metrics. A VIF value exceeding 5.0 or a tolerance value below 0.20 was pre-specified as indicating problematic multicollinearity. Statistical significance was set at *p* < 0.05.

### 2.5. Ethical Considerations

This study was conducted after receiving approval from the Biomedical Research Ethics Committee of Kien An Hospital, Hai Phong, Vietnam, on 25 April 2025, under Decision No. 364/QĐ-BVKA.

## 3. Results

A total of 324 healthcare workers completed the survey. The cohort showed a distinct female predominance, with females accounting for 78.1% (*n* = 253) of the participants compared to 21.9% (*n* = 71) for males. In terms of age distribution, the largest proportion fell into the 30 to <40 years’ age group (53.4%), followed by 40 to <50 years (23.8%), <30 years (12.3%), and ≥50 years (10.5%).

Regarding professional background, the majority of respondents were nurses (58.6%), followed by physicians (24.1%), technicians (9.6%), midwives (5.9%), and other roles (1.9%). An overwhelming majority worked in clinical departments (87.0%) compared to para-clinical departments (13.0%). Reflecting a high level of professional experience, 62.3% of the healthcare workers reported having been employed for more than 10 years, while 27.2% had 6–10 years and 10.5% had 5 years or less of experience.

In terms of workload, most respondents (81.5%) worked ≤40 h per week, with 14.8% working >40 to <60 h, and a minor portion (3.7%) working ≥60 h. Additionally, a high percentage of the sample (90.1%) confirmed having direct contact with patients. Detailed demographic and professional characteristics of the participants are presented in Table 1.

The analysis of PRR among healthcare workers across the 12 dimensions of PSC revealed a wide variation in perceptions. The highest positive responses were observed in the teamwork-related dimensions, specifically “Teamwork within units” at 98.5% (*n* = 319) and “Teamwork across units” at 98.1% (*n* = 318), indicating exceptionally strong perceived collaboration. Managerial support also rated highly, with “Manager’s expectations and actions promoting patient safety” yielding a 93.5% (*n* = 303) positive rate.

Conversely, the lowest positive response was found in “Communication openness” at 25.9% (*n* = 84), closely followed by “Non-punitive response to error” at 29.0% (*n* = 94), which highlights potential barriers to open dialogue and a lingering perception of a punitive environment regarding error management. Additionally, the “Staffing” dimension showed a split perception, with fewer than half of the respondents (49.1%, *n* = 159) reporting a positive view. The detailed frequency and positive response percentages for all 12 dimensions of PSC are presented in Table 2.

The comparison of PSC perceptions between physicians (*n* = 78) and nurses & allied staff (*n* = 246) was evaluated across the 12 dimensions. The results (detailed in Table 3) revealed that the two professional groups shared highly similar perceptions across almost all domains, with only one dimension reaching statistical significance.

Specifically, physicians reported a significantly higher PRR than nurses and allied staff for “Feedback and communication about error” (85.8% vs. 75.2%; *p* = 0.04). While a marginal difference was observed regarding “Staffing”, with nurses and allied staff reporting a higher positive perception than physicians (52.0% vs. 39.7%; *p* = 0.06), this did not meet the traditional threshold for statistical significance. No statistically significant differences (*p* > 0.05) were found between the two professional groups across the remaining 10 dimensions, indicating a broad alignment in how safety culture is perceived across different roles at the study hospital.

Multivariate logistic regression analysis was conducted to identify factors independently associated with a positive overall perception of patient safety culture (PSC). Multicollinearity diagnostics confirmed that collinearity among the independent variables was minimal and within acceptable parameters (all VIF values were <2.5, and tolerance values were >0.40), including between age group and years of service at the hospital. Thus, multicollinearity was not problematic in the final regression model. The results (detailed in Table 4) revealed that age was the only participant characteristic significantly associated with overall PSC perceptions.

After adjusting for other variables, healthcare workers in the 30 to 40 years’ age group (AOR = 4.17, 95%CI: 1.33–13.07, *p* = 0.02) and those in the 40 to 50 years’ age group (AOR = 5.03, 95%CI: 1.30–19.46, *p* = 0.02) demonstrated significantly higher odds of reporting positive perceptions of PSC compared to the younger reference group (<30 years). Conversely, healthcare workers aged over 50 years did not show a statistically significant difference in perception compared to the youngest cohort (AOR = 3.39, 95%CI: 0.80–14.43, *p* = 0.09).

No statistically significant associations (*p* > 0.05) were observed between overall PSC perceptions and any of the other analyzed demographic or professional factors, including gender, professional title, years of service at the hospital, weekly working hours, department type, or direct contact with patients.

## 4. Discussion

The overall positive perception of Patient Safety Culture (PSC) dimensions at the study hospital revealed a wide variation across different domains, highlighting distinct organizational strengths as well as critical areas for systemic improvement. The analysis revealed marked variation across the dimensions: the highest positive response rates were observed in the teamwork-related domains, signaling significant strengths in institutional collaboration. Conversely, the critical dimension of “Communication Openness” recorded the absolute lowest positive rate, closely followed by “Non-punitive response to error”, pointing to a substantial perception gap regarding communication transparency and systemic fairness.

Comparative statistical analysis identified that the previously observed sharp disparities between professional roles were largely absent; physicians and nurses & allied staff demonstrated broad alignment across 11 out of the 12 dimensions. The sole exception was “Feedback and communication about error”, where physicians reported a significantly higher positive perception than nurses and allied staff. Furthermore, the multivariable logistic regression analysis fundamentally reshapes the understanding of individual drivers of PSC within this cohort. Contrary to older models where direct patient contact or department drove perceptions, age emerged as the single dominant predictor of overall PSC, with mid-career healthcare workers demonstrating significantly higher odds of reporting favorable safety culture perceptions compared to their younger counterparts (<30 years).

### 4.1. High-Performing PSC Dimensions

The dimension “Teamwork within units” received an exceptionally high PRR of 98.5% (*n* = 319), closely mirrored by “Teamwork across units” at 98.1% (*n* = 318). The exceptionally high positive response rates observed in this cohort—notably outpacing Western and regional benchmarks—may partly reflect the unique intersection of Vietnam’s rigorous administrative processes and cultural norms. From a managerial standpoint, the establishment of dedicated Quality Management Departments under national mandates ensures that patient safety protocols are non-discretionary components of daily workflows. Unit leaders are directly accountable for safety metrics, which heavily dictate the high scores in “Manager’s expectations and actions promoting patient safety” (93.5%, *n* = 303).

Furthermore, public general hospitals in Vietnam, such as Kien An Hospital, rely on highly centralized, collective clinical structures where staff are organized into rigid, peer-monitored sub-units. This collective work framework naturally fosters high baseline scores in localized teamwork. However, international readers must interpret these highly elevated percentages with a degree of methodological caution. In high-power-distance, hierarchical institutional structures, high agreement rates on administrative surveys might potentially be associated with collective conformity and deference to organizational policy. This structural dynamic can artificially elevate positive perception rates on paper, masking a hidden reluctance to openly question authority or vocalize safety concerns during active clinical workflows. This rate is higher than or comparable to those reported in previous studies conducted in Vietnam (94.2%) [11], the United States (72.0%) [8], El-Jardali’s study (78.5%) [12], and Taiwan (94.0%) [13]. This result aligns with a common finding across both international and Vietnamese research, which consistently identifies effective internal teamwork as a fundamental strength of PSC. This strong internal consensus suggests that, regardless of the specific healthcare context, personnel within the same department or unit highly value collaboration.

The PRR for “Supervisor/manager expectations and actions promoting patient safety” was also very high at 93.5% (*n* = 303). This score is significantly greater than the findings from similar studies such as the United States (75.0%) [8], El-Jardali’s study (79.6%) [12] and Taiwan (84.0%) [13]. These consistently strong findings across diverse cultural settings suggest that there are no substantial cross-cultural differences in the recognized importance of leadership commitment regarding PSC. Both globally and in the Vietnamese context, leaders’ supportive attitudes and clear expectations toward their staff are essential not only for guiding the creation of a safe working environment but also for serving as a vital source of motivation and encouragement.

“Organizational learning—continuous improvement” received a PRR of 87.7% (*n* = 284). This rate is lower than that reported by Xiyao Zhong et al. (92.9%) [14] but significantly higher than the 2023 study conducted in Palestinian hospitals (55.8%) [15]. These robust results indicate a proactive environment where departments and units within the hospital are likely to be actively implementing patient safety assurance activities. Furthermore, this score reflects a culture of systematic learning, where staff are encouraged to continuously learn from errors and evaluate the effectiveness of corrective actions, thereby reinforcing ongoing improvement in patient safety practices.

The dimension “Feedback and communication about error” received a PRR of 77.8% (*n* = 252), which is notably higher than the 2018 AHRQ database rate of 69.0% [8]. This strong finding reflects a high level of comfort and transparency among healthcare workers at Kien An Hospital—Hai Phong, Vietnam, when sharing and discussing incident-related information with their unit leaders. Crucially, the data suggest that staff are regularly informed and updated by unit leaders about reported events, and team discussions are actively held to develop corrective and preventive actions aimed at avoiding recurrence. The robust exchange of information within departments demonstrates a well-functioning feedback loop, which is a key component of a sustainable PSC and should be further encouraged and institutionalized.

In contrast to the strong feedback mechanism, “Communication Openness” received a significantly lower PRR of 25.9% (*n* = 84). This result is particularly noteworthy as numerous studies on PSC, both globally and in Vietnam, have consistently identified “Communication Openness” as one of the weakest dimensions across most hospital settings. For instance, Hefner (2017) reported a PRR of 49.0% [16], while Xiyao Zhong et al. at Peking University Hospital, China, found a rate of 52.2% [14].

The lack of openness in communication, as Xiyao Zhong noted, poses major challenges to patient safety during healthcare delivery [14]. Several factors may contribute to this pervasive weakness, especially within an Asian context. I-Chi Chen suggested that a general fear of making mistakes and a strong cultural desire to maintain harmony often lead individuals to avoid open discussions about errors, resulting in less objective evaluations [13]. Additionally, cultural characteristics prevalent in many Asian settings, where employees tend to adhere strictly to hierarchical norms, may contribute to this challenge. In such environments, reporting an error that occurs within a department may be perceived as “whistleblowing”, which consequently discourages open communication about mistakes and safety risks.

The PRR for the “Staffing” dimension was 49.1% (*n* = 159), which is lower than the results reported in studies conducted at Peking University Hospital, China (53.7%) [14] but notably higher than at Palestine Hospital (2023) (34.8%) [15]. Since human resources are a crucial component contributing significantly to the healthcare system’s capacity, this result suggests that fewer than half of the respondents view current staffing levels favorably. Considering that work overload due to insufficient staffing can lead to fatigue, stress, and compromised patient care quality, this dimension should be treated as a critical early warning indicator of potential patient safety risks, necessitating the implementation of proactive and long-term workforce planning strategies.

The assessment of leadership commitment and organizational coordination also revealed high positive scores. “Hospital Management Support for Patient Safety” achieved 70.7% (*n* = 229), though lower than rates reported in China by Xiyao Zhong et al. (83.7%) [14], it aligns closely with the U.S. by Famolaro et al. (72.0%) [8]. This highlights that sustained leadership commitment plays a vital role in fostering a robust PSC. The high response rate suggests that leaders at both departmental and hospital levels actively provide professional guidance and motivational support, which successfully promotes a sense of security and confidence among staff.

Similarly, “Teamwork Across Hospital Units” received a PRR of 98.1% (*n* = 318), significantly outperforming the AHRQ 2018 benchmark (62.0%) [8], the Palestine study (63.1%) [15], and the Chinese study (89.7%) [14]. This finding confirms that effective inter-unit collaboration is a recognized organizational strength. Given that patient safety in complex healthcare systems relies heavily on seamless collaboration and coordination among multidisciplinary teams, this result reflects a positive organizational climate that actively fosters mutual support, communication, and shared responsibility across different departments.

In sharp contrast to the high-scoring organizational dimensions, “Non-punitive Response to Error” recorded a low PRR of 29.0% (*n* = 94). Although this score is numerically lower than several international benchmarks, such as AHRQ (47.0%) [8] nd Xiyao Zhong et al. (51.1%) [14], it aligns closely with a U.S. study (28.0%) [16]. The low score for “Non-punitive response to error” aligns with studies from other Asian hospital settings [14,15], indicating that transition from a traditional blame culture toward a system-oriented learning culture remains an ongoing challenge. The critically low scores observed in both ‘Communication Openness’ and ‘Non-punitive response to error’ dimensions highlight a profound systemic challenge regarding institutional trust and organizational learning at Kien An Hospital. In many traditional healthcare environments, errors are historically treated as individual failures rather than systemic vulnerabilities. When staff perceive that mistakes are met with personal blame, administrative penalties, or professional reputational damage, a culture of defensive concealment inevitably emerges. This fear of retribution directly suppresses the reporting of near-misses and minor adverse events. From an organizational learning perspective, this reporting silence acts as a critical barrier; it deprives hospital leadership of the vital data required to conduct comprehensive root-cause analyses and implement structural modifications. To transition from a culture of individual culpability to an enlightened safety culture, hospital leadership must actively champion psychological safety. This requires decoupling incident reporting from punitive disciplinary actions, thereby building the institutional trust necessary to transform errors into valuable opportunities for continuous systemic improvement.

### 4.2. Some Factors Influencing PSC

#### 4.2.1. The Dimension of PSC Between Physicians and Nurses & Allied Staff

The comparison between professional strata revealed remarkable alignment across almost all dimensions of patient safety culture, with only one dimension reaching statistical significance. For “Feedback and communication about errors”, both groups reported relatively high PRR, but the positive response rate of physicians in this domain was significantly higher than that of nurses and allied staff (85.8% vs. 75.2%; *p* = 0.04). This difference is noteworthy, as understanding the causes of errors and learning from them is an essential approach in continuous quality improvement [17]. Furthermore, professional development literature suggests that younger staff can learn invaluable lessons from more experienced colleagues through collaborative practice [18] Therefore, these differences highlight a specific opportunity for individual physicians, professional teams, and hospital managers to leverage the current strengths in feedback mechanisms to enhance and strengthen the overall PSC.

Conversely, regarding the “Overall perceptions of patient safety” dimension, the positive response rates between physicians and nurses & allied staff were statistically similar (76.9% vs. 74.3%; *p* = 0.65). This broad alignment across professional lines contrasts with older comparative studies conducted in public provincial settings in Vietnam, such as that by Tran et al. [19], which identified lower PSC perceptions among nursing staff compared to physicians. This shared perspective can be understood through the lenses of clinical accountability and differing workflow configurations. Nursing and allied staff typically operate within highly standardized, collaborative, and peer-supported clinical pathways. Their structured routines and continuous shift handovers reinforce a sense of localized process reliability and safety net protection. Meanwhile, physicians bear the ultimate, individualized legal and clinical liability for final diagnostic and therapeutic outcomes. This acute burden of clinical accountability, paired with a workflow characterized by high-stakes autonomy, often makes physicians highly sensitive to organizational gaps or communication breakdowns. In this study, both groups evaluated overall safety climate metrics through a statistically equivalent framework.

#### 4.2.2. Individual Factors Influencing PSC

In the multivariable model, age was the sole factor independently associated with favorable PSC perceptions. Compared to staff aged <30 years, healthcare workers aged 30–40 years (AOR = 4.17) and 40–50 years (AOR = 5.03) exhibited significantly higher odds of reporting positive safety culture perceptions. This pattern may be linked to professional developmental stages. Junior healthcare professionals (<30 years) work directly in high-volume patient delivery where system vulnerabilities (e.g., staffing bottlenecks, supply delays) are acutely felt, potentially leading to lower safety climate ratings. As staff mature into mid-career roles, increased clinical familiarity, higher institutional status, and greater navigation mastery may contribute to more favorable perceptions of safety systems [16]. Because safety perceptions were broadly shared across professional roles and departments, quality improvement interventions should focus on early-career onboarding and fostering psychological safety hospital-wide rather than targeting isolated departments.

This pronounced age-associated trend highlights critical professional development dynamics. Early-career healthcare workers (<30 years) operate at the sharp end of medicine, where systemic vulnerabilities—such as staffing shortages, equipment delays, and fragmented communication—manifest as immediate, real-time hazards. Encountering high cognitive workloads while adapting to a new institutional culture strips away an idealized view of safety policies, causing younger staff to perceive the safety climate less favorably. As healthcare workers mature into mid-career cohorts (30–50 years), they gain substantial clinical competence, achieve higher status within the hospital hierarchy, and adapt to organizational navigation, resulting in a more confident and favorable outlook on institutional safety frameworks.

Crucially, the fact that factors like direct patient contact (AOR = 0.47, *p* = 0.29), professional title (AOR = 1.04, *p* = 0.91), or years of service (*p* = 0.12) were non-significant in the multivariable model indicates that the hospital’s safety climate challenges are macro-institutional rather than confined to specific departments or roles. Young staff across all professional strata experience the safety culture less favorably, signaling to hospital policymakers that safety initiatives must be co-designed with a focus on junior personnel, targeting onboarding protocols and building psychological safety. This result stands in contrast to the findings of Nguyen Thi Huong’s study at Viet Nam–Cuba Friendship Hospital (2020), where staff with direct patient contact reported a higher positive response rate [11], reinforcing that safety culture determinants remain highly setting-specific.

### 4.3. Study Limitations

Several limitations should be considered when interpreting the results of this cross-sectional study. First, the study was conducted at a single hospital setting with a sample size of 324 healthcare workers, which may limit the generalizability of the findings to the broader population of Vietnamese hospitals.

Second, the use of a self-reported questionnaire method carries an inherent risk of response bias. Within a high-power-distance cultural environment, social desirability bias may have led participants to over-report positive safety culture perceptions, particularly in sensitive areas, thereby potentially inflating the positive response rates across administrative or managerial dimensions.

Third, the instrument itself presents a potential limitation. While a minimum Cronbach’s alpha of 0.62 meets the threshold for acceptable internal consistency, it falls marginally below standard AHRQ reference benchmarks, indicating slightly reduced response reliability within that specific dimension. This reduced consistency—potentially driven by participant cognitive burden when interpreting reverse-coded items—introduces minor measurement error that should be considered when interpreting that domain’s findings.

## 5. Conclusions

This study establishes a vital baseline assessment of the Patient Safety Culture (PSC) at the study hospital, highlighting prominent operational strengths alongside deep structural vulnerabilities. While institutional strong points like “Teamwork within units” and “Teamwork across units” demonstrate a highly resilient baseline of peer collaboration, the exceptionally low positive response rates in “Communication Openness” and “Non-punitive response to error” indicate an urgent, systemic need for administrative reform. These findings signal that while staff collaborate exceptionally well within established clinical protocols, a prevailing “blame culture” and hierarchical barriers actively suppress open dialogue and the transparent reporting of errors.

**Practical Recommendations**

- Based on the significant findings regarding age-driven perceptions and low communication openness, the following strategic interventions are recommended for hospital leadership.
- Target Early-Career Personnel: Since healthcare workers under 30 years old perceive the safety climate least favorably, hospital administration should design targeted onboarding protocols and mentorship programs focused on navigating safety systems.
- Cultivate Psychological Safety: Leadership must actively decouple clinical error reporting from punitive disciplinary actions or administrative penalties to dismantle the lingering “blame culture.”
- Enhance Communication Openness: Implement transparent, non-punitive reporting channels and structured feedback loops that encourage junior staff to voice safety concerns without fear of professional or reputational retribution.
- Institutionalize Systemic Learning: Shift the organizational focus from individual culpability to comprehensive root-cause analysis, transforming active errors into collective opportunities for continuous quality improvement.

## Figures and Tables

**Table 1:** Personal characteristics of the study participants by groups (*n* = 324).

No.	Characteristics	Group	Frequency	Percentage (%)
1	Gender	Male	71	21.9
		Female	253	78.1
2	Age group	<30 years	40	12.3
		30–<40 years	173	53.4
		40–<50 years	77	23.8
		≥50 years	34	10.5
3	Working department	Clinical	282	87.0
		Para-clinical	42	13.0
4	Job title	Physician	78	24.1
		Nurse	190	58.6
		Technician	31	9.6
		Midwife	19	5.9
		Others	6	1.9
5	Years of experience	≤5	34	10.5
		6–10	88	27.2
		>10	202	62.3
6	Working hours/week	≤40	264	81.5
		>40–<60	48	14.8
		≥60	12	3.7
7	Patient contact	Yes	292	90.1
		No	32	9.9

**Table 2:** Frequency and percentage of positive PRR on PSC at study hospital (incorporating reverse-coded metrics for all negatively worded items).

PSC Dimension	*n* (%)
1. Teamwork within units	319 (98.5)
2. Manager’s expectations and actions promoting patient safety	303 (93.5)
3. Organizational learning—continuous improvement	284 (87.7)
4. Feedback and communication about error	252 (77.8)
5. Communication openness	84 (25.9)
6. Staffing	159 (49.1)
7. Non-punitive response to error	94 (29.0)
8. Hospital management support for patient safety	229 (70.7)
9. Teamwork across units	318 (98.1)
10. Handoffs and transitions	261 (80.1)
11. Frequency of event reporting	201 (62.0)
12. Overall perceptions of patient safety	243 (75.0)

**Table 3:** PRR comparison of PSC Dimensions between Physicians (*n* = 78) and Nurses & Allied staff (246).

PSC Dimension	Physicians		Nurses & Allied Staff		*p*
	**PRR**	**(%)**	**PRR**	**(%)**	
1. Teamwork within units	78	100	241	97.9	0.20
2. Manager’s expectations and actions promoting patient safety	74	94.8	229	93.1	0.57
3. Organizational learning—continuous improvement	67	85.8	217	88.2	0.58
4. Feedback and communication about error	67	85.8	185	75.2	**0.04**
5. Communication openness	19	24.3	65	26.4	0.71
6. Staffing	31	39.7	128	52.0	0.06
7. Non-punitive response to error	20	25.6	74	30.1	0.45
8. Hospital management support for patient safety	53	67.9	176	71.5	0.54
9. Teamwork across units	76	94.4	242	98.3	0.59
10. Handoffs and transitions	66	84.6	195	79.2	0.29
11. Frequency of event reporting	46	58.9	155	63.0	0.52
12. Overall perceptions of patient safety	60	76.9	183	74.3	0.65

**Table 4:** Association Between Overall PSC Factors and Participants’ Characteristics.

Variables	Overall Patient Safety Culture		Crude OR (95% CI)	*p* (Univariable)	Adjusted OR (95% CI)	*p* (Multivariable)
	Positive, *n* (%)	Not Positive, *n* (%)				
	Gender					
Male	52 (73.2)	19 (26.8)	1.00 (ref)		1.00 (ref)	
Female	194 (76.7)	59 (23.3)	1.20 (0.66–2.19)	0.55	1.31 (0.68–2.53)	0.42
	Age group					
<30 years	26 (65.0)	14 (35.0)	1.00 (ref)		1.00 (ref)	
30–40 years	136 (78.6)	37 (21.4)	1.98 (0.94–4.17)	0.07	4.17 (1.33–13.07)	0.02
40–50 years	60 (77.9)	17 (22.1)	1.90 (0.82–4.42)	0.13	5.03 (1.30–19.46)	0.02
>50 years	24 (70.6)	10 (29.4)	1.29 (0.48–3.45)	0.61	3.39 (0.80–14.43)	0.09
	Professional title					
Doctor	59 (75.6)	19 (24.4)	1.00 (ref)		1.00 (ref)	
Nurse & Allied staff	187 (76.0)	59 (24.0)	1.02 (0.56–1.85)	0.94	1.04 (0.52–2.07)	0.91
	Years of service at the hospital					
<5 years	25 (73.5)	9 (26.5)	1.00 (ref)		1.00 (ref)	
6–10 years	69 (78.4)	19 (21.6)	1.31 (0.52–3.27)	0.56	0.54 (0.16–1.90)	0.34
>10 years	152 (75.2)	50 (24.8)	1.09 (0.48–2.50)	0.84	0.33 (0.08–1.36)	0.12
	Working hours/week					
≤40 h	198 (75.0)	66 (25.0)	1.00 (ref)		1.00 (ref)	
>40–<60 h	38 (79.2)	10 (20.8)	1.27 (0.60–2.68)	0.53	1.31 (0.60–2.87)	0.49
≥60 h	10 (83.3)	2 (16.7)	1.67 (0.36–7.80)	0.52	2.08 (0.42–10.23)	0.36
	Department					
Clinical	214 (75.9)	68 (24.1)	1.00 (ref)		1.00 (ref)	
Para-clinical	32 (76.2)	10 (23.8)	1.02 (0.48–2.18)	0.96	1.83 (0.49–6.85)	0.37
	Contact with patients					
Yes	223 (76.4)	69 (23.6)	1.00 (ref)		1.00 (ref)	
No	23 (71.9)	9 (28.1)	0.79 (0.35–1.79)	0.57	0.47 (0.11–1.92)	0.29

## Data Availability

The data presented in this study are available on request from the corresponding author. The data are not publicly available due to privacy and ethical restrictions regarding the confidentiality of the healthcare professionals who participated in the survey.

## Appendix A. HSOPSC-VN Questionnaire

**Patient Safety Culture Across 12 Domains**		
Abbreviations: LOA = Level of Agreement LOP = Level of Perception/Assessment AF = Assessment Frequency EL = Evaluation Level		
**A. PERCEPTIONS OF YOUR WORK UNIT/DEPARTMENT**		
A1	Mutual support within unit/department	Degree of willingness to support each other’s work when needed.
A2	Staffing in unit/department	Level of Agreement (LOA) regarding having a sufficient number of healthcare staff.
A3	Teamwork to complete tasks	LOA on coordinating as a team within the unit to accomplish work when quick completion is required.
A4	Mutual respect	LOA regarding mutual respect among all personnel in the unit/department.
A5	Working hours	LOA regarding working hours, specifically the perception that working longer than standard hours is required to complete tasks.
A6	Ensuring patient safety	LOA regarding patient safety assurance activities within the unit/department.
A7	Use of additional temporary/external staff	LOA on the unit needing additional healthcare staff (non-permanent/external) to ensure patient care.
A8	Facing prejudice/stigma when errors occur	Level of Perception/Assessment (LOP) regarding healthcare staff facing prejudice or negative bias when errors occur.
A9	Quality improvement from past errors	LOP regarding the unit achieving better quality improvements based on past errors.
A10	Absence of errors attributed to luck	LOP regarding the perception that the absence of errors in the unit is due to luck rather than proactive prevention.
A11	Support between sections within the same department	LOP regarding different sub-units/sections within the department supporting each other when needed.
A12	Individual blame when incidents occur	LOP regarding assigning individual blame when an incident occurs without investigating process or system failures.
A13	Post-intervention evaluation	LOP regarding evaluating effectiveness after implementing quality improvement measures (interventions).
A14	Rushing to complete work	LOP regarding staff working hastily or rushing to finish tasks.
A15	Prioritizing patient safety	LOP regarding the unit always prioritizing patient safety over rushing to finish work quickly.
A16	Recording errors in personnel files	LOP regarding staff worry that errors will be documented in their personal personnel files.
A17	Patient safety concerns	LOP regarding the unit experiencing issues that compromise patient safety.
A18	Error prevention measures	LOP regarding the unit having established procedures and measures to prevent errors from occurring.
**B. PERCEPTIONS OF UNIT/DEPARTMENT LEADERSHIP**		
B1	Encouragement and recognition for safety compliance	LOA regarding unit leadership encouraging and motivating staff when adhering to standard protocols and ensuring patient safety.
B2	Acknowledging safety improvement suggestions	LOA regarding unit leadership consistently considering and acknowledging staff suggestions for patient safety improvement.
B3	Pressuring staff under high workload	LOA regarding unit leadership pressuring staff to work faster—including skipping steps—when workload increases.
B4	Disregard for patient safety issues	LOA regarding unit leadership ignoring patient safety issues despite knowing that errors recur repeatedly.
**C. COMMUNICATION WITHIN THE UNIT/DEPARTMENT**		
C1	Feedback on error-driven improvement measures	Assessment Frequency (AF) regarding staff receiving feedback on patient safety improvements implemented based on reported errors.
C2	Communicating safety concerns to unit leadership	AF regarding staff feeling comfortable discussing issues that negatively impact patient safety with leadership.
C3	Information sharing on error incidents	AF regarding staff being informed about error incidents that occurred in the unit/department.
C4	Input on patient safety decisions	AF regarding staff feeling comfortable discussing patient safety decisions with unit leadership.
C5	Conducting discussions to prevent error recurrence	AF regarding the unit organizing discussion sessions on measures to prevent error recurrence.
C6	Hesitation to speak up or ask questions	AF regarding staff feeling hesitant or afraid to speak up/ask questions when noticing incorrect practices.
**D. EVENT REPORTING FREQUENCY**		
D1	Reporting frequency for near-miss errors	Evaluation Level (EL) on reporting frequency when a near-miss occurs or an error is intercepted before affecting the patient.
D2	Reporting frequency for no-harm errors	EL on reporting frequency when an incident occurs but causes no harm to the patient.
D3	Reporting frequency for errors with potential/uncertain harm	EL on reporting frequency when an incident occurs where potential harm to the patient is uncertain.
**E. PATIENT SAFETY GRADE IN THE UNIT/DEPARTMENT**		
E1	Overall patient safety grade	Overall assessment of the grade/quality of patient safety in the current work unit/department.
**F. PERCEPTIONS OF THE HOSPITAL**		
F1	Patient safety cultural climate	EL regarding hospital leadership creating a work environment oriented toward patient safety.
F2	Lack of inter-departmental coordination	EL regarding poor coordination between different hospital departments.
F3	Patient transfers between departments	EL regarding vital handover information being missed when transferring patients between departments.
F4	Collaboration among affiliated departments	EL regarding effective coordination and collaboration among related departments.
F5	Shift handover of patient information	EL regarding important patient information being omitted during shift-to-shift handovers.
F6	Comfort level in working with staff from other departments	EL regarding feeling uncomfortable when communicating/collaborating with personnel from other departments.
F7	Communication issues between departments	EL regarding frequent problems arising during inter-departmental information exchange.
F8	Patient safety as a top organizational priority	EL regarding hospital management placing patient safety as the highest priority.
F9	Leadership focus limited to severe incidents	EL regarding hospital leadership only paying attention to patient safety when serious errors occur.
F10	Effective inter-departmental collaboration	EL regarding departments collaborating effectively to ensure optimal patient care.
F11	Shift incident & workflow coordination	EL regarding inter-departmental teamwork during shifts to ensure patient care quality.

## References

[1] Fekadu G., Muir R., Tobiano G., Bime A.E., Ireland M.J., Marshall A.P. (2025). Patient safety culture in resource-limited healthcare settings: A multicentre survey. PLoS ONE.

[2] World Patients Alliance WHO Consultation on Global Patient Safety Action Plan. https://www.worldpatientsalliance.org/news/who-consultation-on-global-patient-safety-action-plan/.

[3] Agency for Healthcare Research and Quality (2013). National Scorecard on Hospital-Acquired Conditions.

[4] Ngo H.A., Pham T.T., Bui T.T.Q. (2022). Current situation and factors affecting medical incident management at District 2 Hospital, Ho Chi Minh City, Vietnam, in 2021. J. Health Dev. Stud..

[5] Institute of Medicine (2000). To Err is Human: Building a Safer Health System.

[6] Nieva V.F., Sorra J. (2003). Safety culture assessment: A tool for improving patient safety in healthcare organizations. Qual. Saf. Health Care.

[7] Arabloo J., Rezapour A., Ebadi Fard Azar F., Mobasheri Y. (2012). Measuring patient safety culture in Iran using the Hospital Survey on Patient Safety Culture (HSOPS): An Exploration of Survey Reliability and Validity. Int. J. Hosp. Res..

[8] Agency for Healthcare Research and Quality (2018). Hospital Survey on Patient Safety Culture: 2018 User Database Report.

[9] Hoffmann B., Müller V., Rochon J., Gondan M., Müller B., Albay Z., Weppler K., Leifermann M., Mießner C., Güthlin C. (2014). Effects of a team-based assessment and intervention on patient safety culture in general practice: An open randomised controlled trial. BMJ Qual. Saf..

[10] Thuong T.C., Hung N.T., Lien L.B., Hieu D.T., Niem D.V., Minh N.N.Q., Le N.T.C., Nha N.V.T., Nhung D.B.T. (2012). Hospital Survey On Patient Safety Culture In Children’s Hospital 1 In 2012. Ho Chi Minh City J. Med..

[11] Huong N.T., Hoa P.T.M., Nhu H.V. (2021). Current status and factors influencing patient safety culture among healthcare workers at Vietnam–Cuba Friendship Hospital 2020-cohort. J. Health Dev. Stud..

[12] El-Jardali F., Sheikh F., Garcia N.A., Jamal D., Abdo A. (2014). Patient safety culture in a large teaching hospital in Riyadh: Baseline assessment, comparative analysis and opportunities for improvement. BMC Health Serv. Res..

[13] Chen I.C., Li H.H. (2010). Measuring patient safety culture in Taiwan using the Hospital Survey on Patient Safety Culture (HSOPSC). BMC Health Serv. Res..

[14] Zhong X., Song Y., Dennis C., Slovensky D.J., Wei L.Y., Chen J., Ji J. (2019). Patient safety culture in Peking University Cancer Hospital in China: Baseline assessment and comparative analysis for quality improvement. BMC Health Serv. Res..

[15] Zabin L.M., Qaddumi J., Ghawadra S.F. (2025). The relationship between job stress and the perception of patient safety culture among Palestinian hospital nurses. BMC Nurs..

[16] Hefner J.L., Hilligoss B., Knupp A., Bournique J., Sullivan J., Adkins E., Moffatt-Bruce S.D. (2017). Cultural transformation after implementation of crew resource management: Is it really possible?. Am. J. Med. Qual..

[17] Bottcher B., Abu-El-Noor N., Abuowda Y., Alfaqawi M., Alaloul E., El-Hout S., Al-Najjar I., Abu-El-Noor M. (2019). Attitudes of doctors and nurses to patient safety and errors in medical practice in the Gaza Strip: A cross-sectional study. BMJ Open.

[18] Azyabi A., Karwowski W., Davahli M.R. (2021). Assessing patient safety culture in hospital settings. Int. J. Environ. Res. Public Health.

[19] Tran T.N.H., Pham Q.T., Tran L.H., Vu T.A., Nguyen M.T., Pham H.T., Le T.T., Bui T.T.H. (2022). Comparison of perceptions about patient safety culture between physicians and nurses in public hospitals in Vietnam. Risk Manag. Healthc. Policy.

